# Supplementary material for: A human neural crest model reveals the developmental impact of neuroblastoma-associated chromosomal aberrations
Source: Nat Commun. 2024 May 3;15:3745. doi: 10.1038/s41467-024-47945-7 (PMC11068915; doi:10.1038/s41467-024-47945-7)
Supplement: Supplementary file 2 — Supplementary Information [file 41467_2024_47945_MOESM2_ESM.pdf]

A human neural crest model reveals the developmental impact of neuroblastoma-associated chromosomal aberrations

Saldana-Guerrero IM*, Montano-Gutierrez LF*, et al., Nature Communications (2024)

SUPPLEMENTARY FIGURES

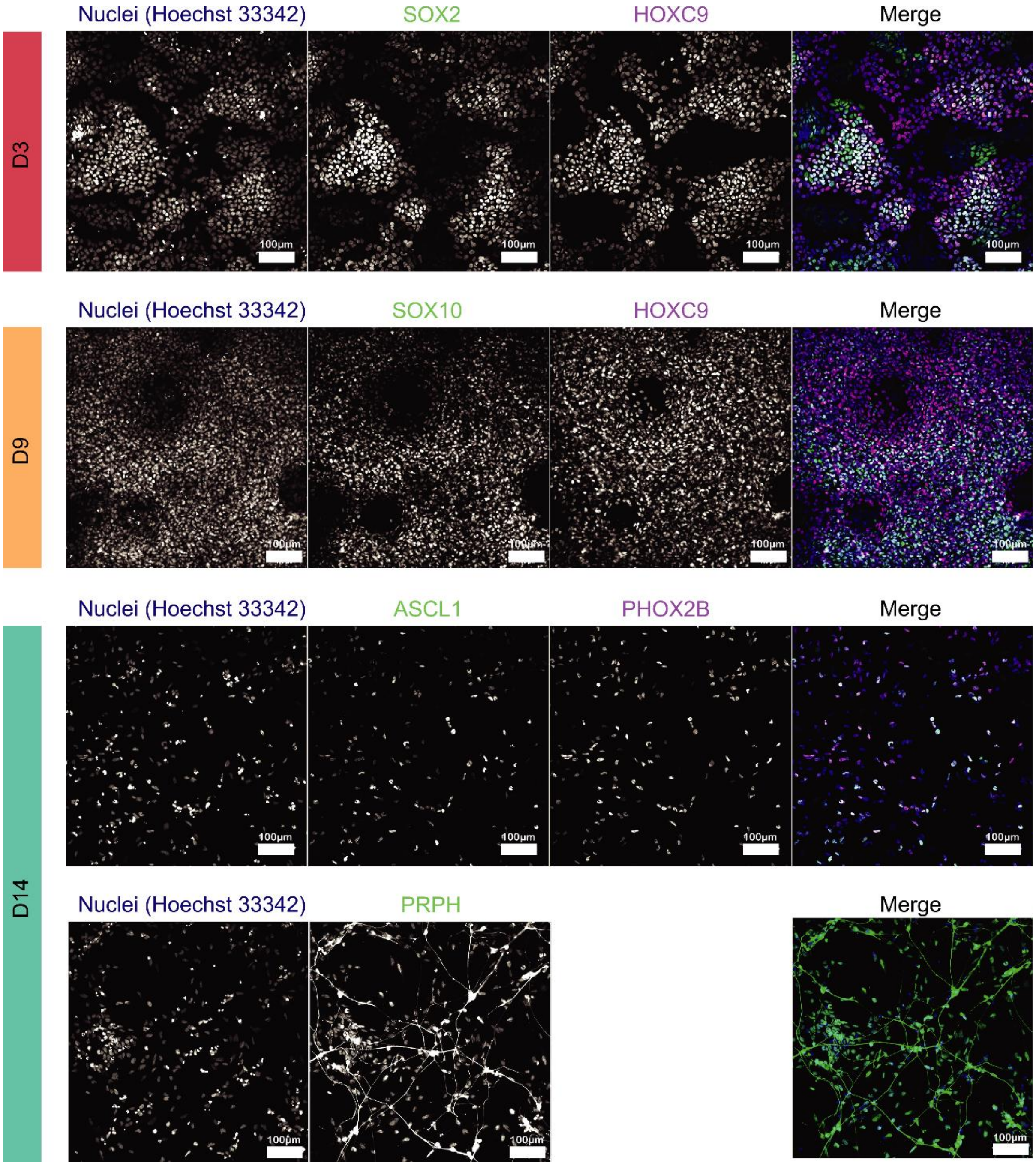

Supplementary Figure 1. Immunofluorescence analysis of hESCs during trunk NC differentiation.
Immunofluorescence analysis of the expression of indicated markers at different time points during the differentiation of hESCs toward trunk NC and its derivatives. All experiments were repeated at least three times with similar results. D3/9/14, day 3/9/14.

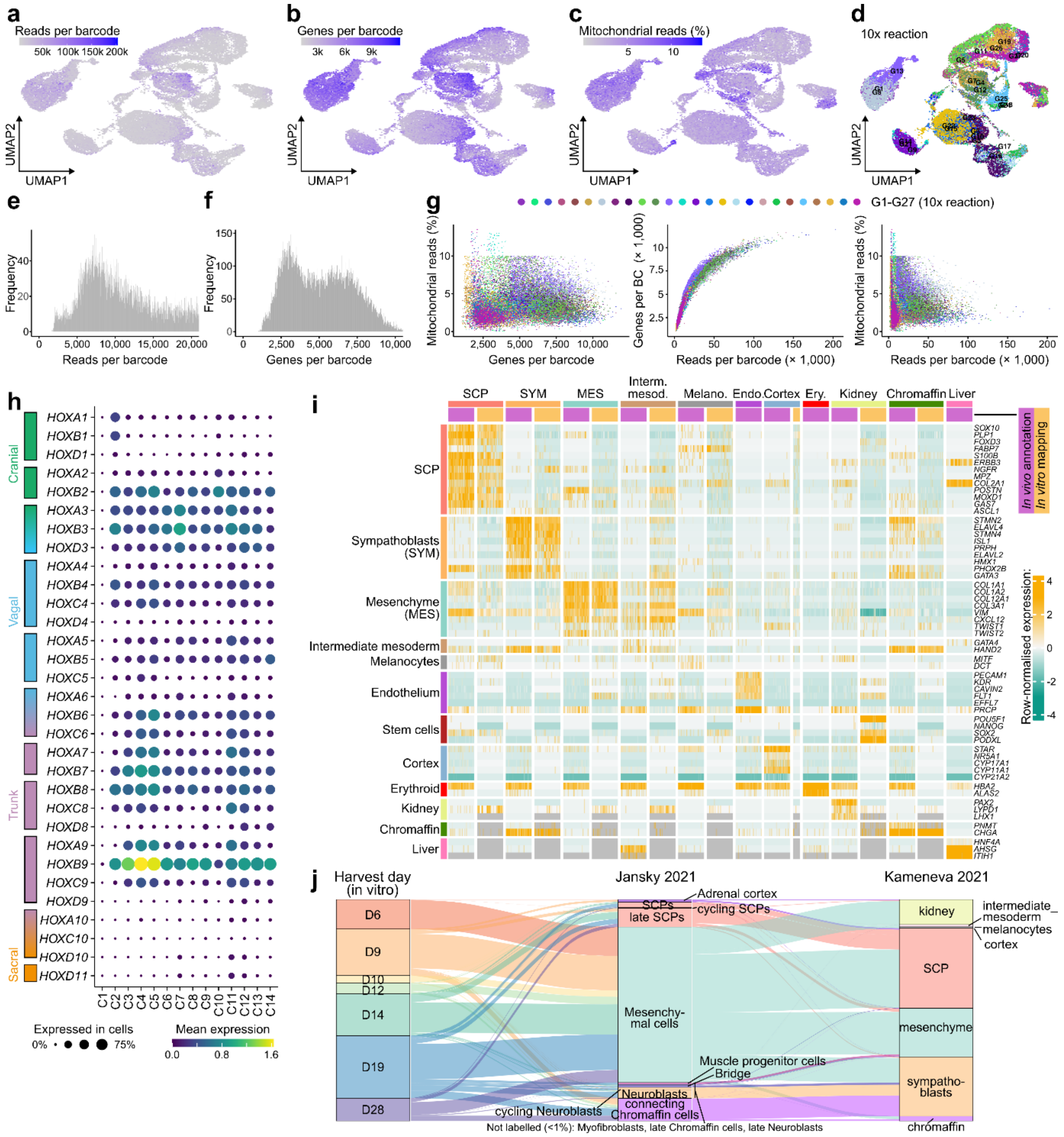

Supplementary Figure 2. Quality control and reference mapping of single-cell RNA-seq data from wild-type hESC trunk neural crest differentiation.

(a-d) UMAP plots showing QC covariates for the WT in-vitro dataset in Figure 1. **(e-f)** Histograms depicting the distribution of read depth (e) and number of genes detected (f) per cell barcode after QC. **(g)** Scatterplots comparing all QC covariates (panels a-c) per cell, coloured by 10x Genomics run (G1-G27; cp. Fig. 1b). **(h)** Bubble plot indicating the mean expression (colour) and fraction of cells expressing (size) HOX genes per cluster. Genes have been ordered from cranial to sacral axis specification. **(i)** Side-by-side comparison of cell-type marker expression (rows) in the human adrenal gland reference[1] versus cells mapped to the same cell types in our WT in-vitro dataset. In each case, 200 cells (columns; divided by cell type and then dataset) were selected randomly for display purposes. Values are depth-normalised per experiment and row-scaled globally. Known stem cell markers were added to trace the mapping of our in-vitro hESCs to the reference (which does not contain ESC-like cells); in this case, this was “kidney”. Cells erroneously mapped, absent from the reference,

or lacking relevant cell-type markers were classified as “other” and coloured grey in Figure 1e. **(j)** Alluvial plots comparing the mappings of cells in the in-vitro dataset to two adrenal gland references[1,2]. Each “stream” indicates a group of cells mapped to one cell type in the Jansky et al. (middle) and Kameneva et al. (right) references (also indicated in colour). For example, cells that were labelled MES in Kameneva et al. (used to define cell type labels in this paper) also mapped to mesenchymal cells in the Jansky et al. reference, cells that mapped to SYM mapped to cycling neuroblasts and to neuroblasts. Cells that we labelled as SCPs split into cells that mapped to mesenchymal cells and late SCPs in Jansky et al., consistent with our observations that some “SCP-like” cells in our dataset represent a less mature, early progenitor state (see main text). Source data are provided as a Source Data file. QC, quality control; UMAP, Uniform Manifold Approximation and Projection; SCP, Schwann cell precursor; SYM, sympathoblast; MES, mesenchymal.

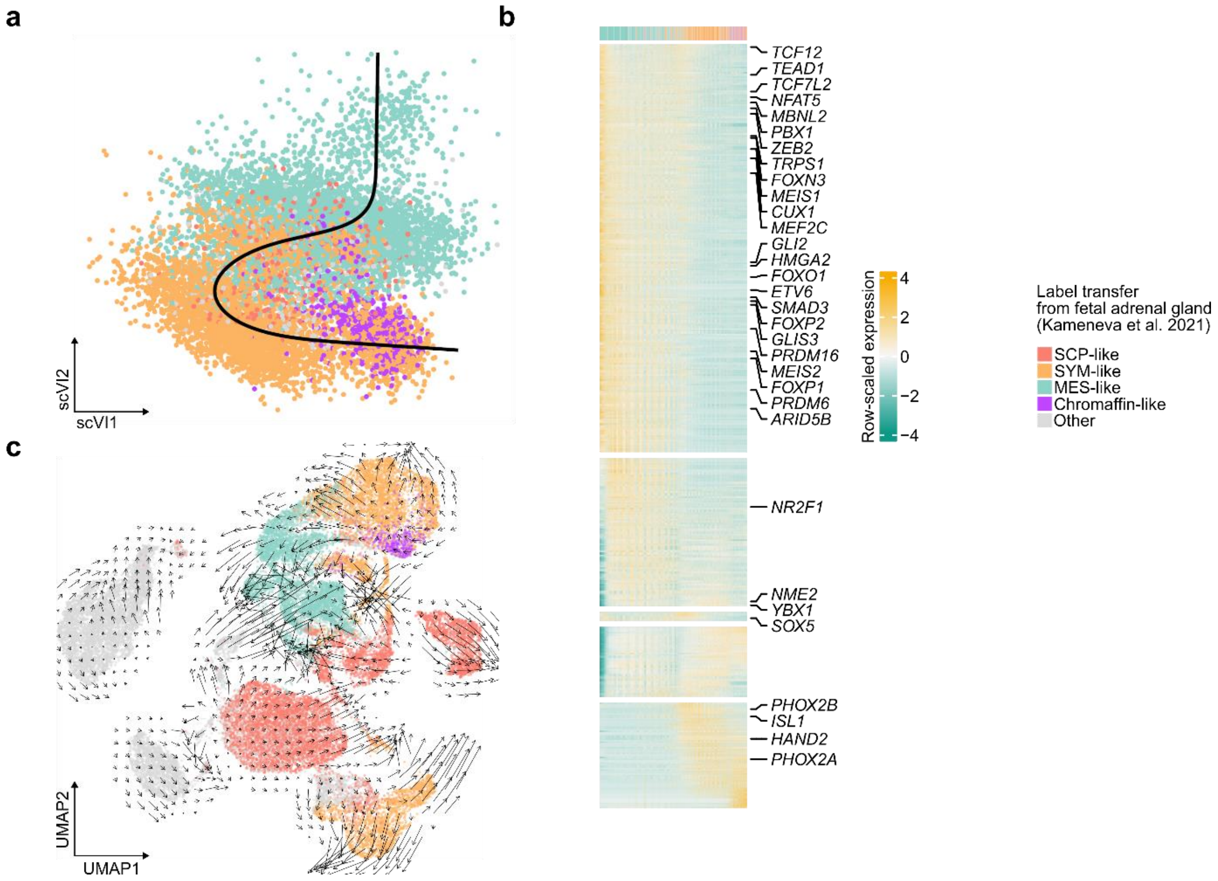

Supplementary Figure 3. Trajectories connecting gradients of transcriptionally similar cells at different developmental stages.

(a) Slingshot[3] pseudotime trajectories (top) for cell clusters C11-C14. Cells were extracted from the main WT reference dataset and reprocessed (see "Basic scRNA-seq processing" in Methods), and trajectories were calculated on the first two scVI components. **(b)** Heatmap showing the top genes with the strongest association with the trajectory as ranked by tradeSeq's Wald test[4] (based on a fitGAM model with 5 knots). Highlighted genes are all the transcription factors found in the association test. Supplementary Data 3 reports all genes found in this analysis. **(c)** RNA velocities calculated for the cells in Figure 1d using Velocyto[5]. WT, wild-type H7 hESCs; SCP, Schwann cell precursor; SYM, sympathoblast; MES, mesenchymal.

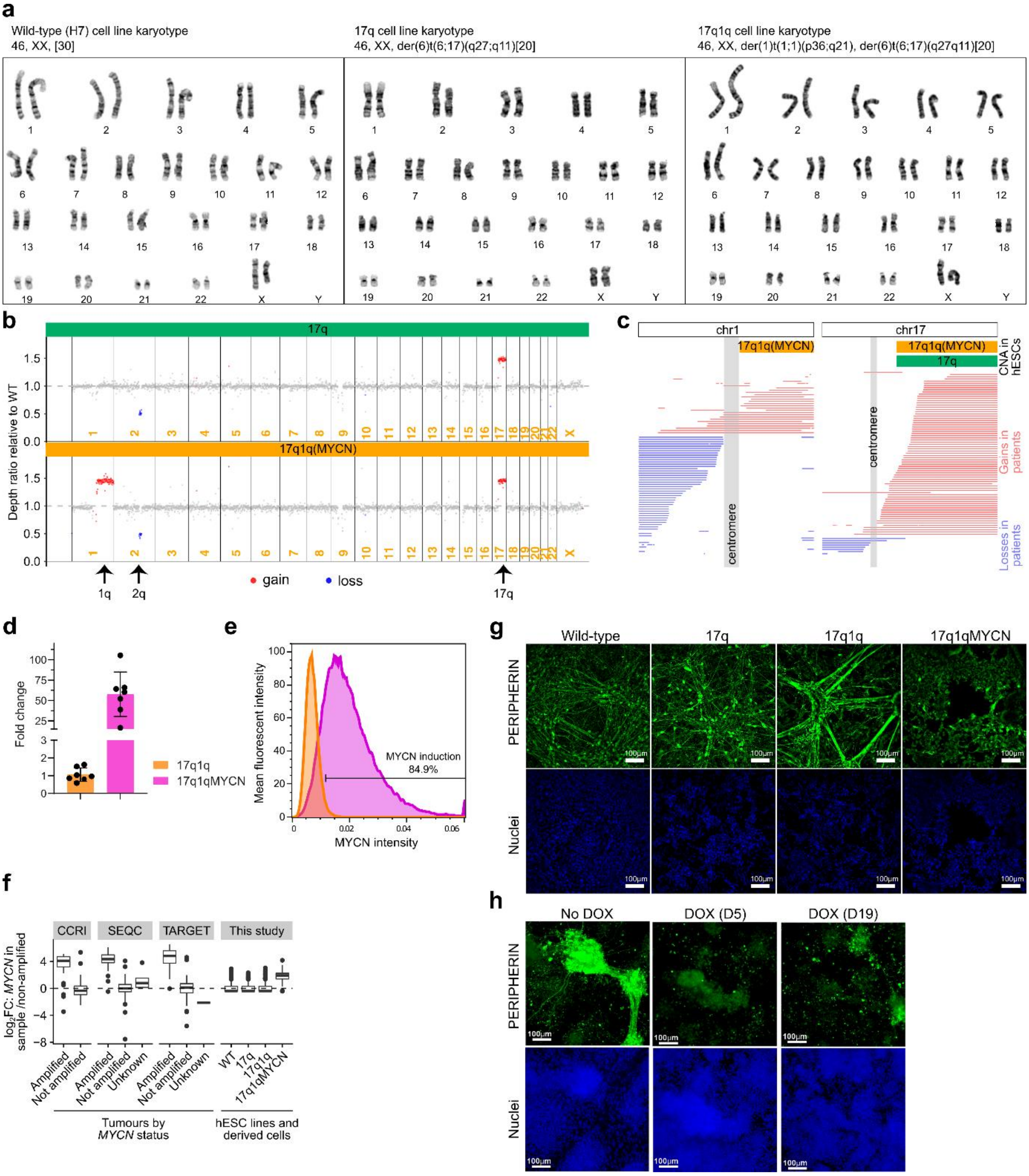

Supplementary Figure 4. Genetic and phenotypic characterization of mutant hESC lines.

(a) Cytogenetic analysis of the H7-derived hESC lines used in the study. **(b)** Plots of the depth ratio calculated between each sample and the parental control using Sequenza[6]. CNAs can be seen for chr1q and chr17q, and a small segment which is lost on chr2 (q23.3-24.3). CNA coordinates and SNVs in these cell lines are reported in Supplementary Data 4 and 5. **(c)** Pile-up of segmental gains (red) and losses (blue) on chromosome 1 (left) and 17 (right) based on SNP array data of 88 NB samples[7] (thin lines) compared to CNAs in our 17q and 17q1q(MYCN) hESC lines (from panel b). The X axis corresponds to chromosome position. Each horizontal line depicts all the aberrations found for the same patient. For compatibility with available annotations, coordinates in this plot are shown with respect to human genome reference GRCh37/hg19 (while all other analysis in this paper were done using

hg38/GRCh38). **(d,e)** Analysis of MYCN expression at the transcript (d) and protein (e) level in D9 17q1qMYCN cultures after DOX treatment at D5 vs untreated control following qPCR and flow cytometry analysis respectively. Bar plots in d show the mean of n = 3 biological replicates (error bars = SEM). All flow cytometry experiments were repeated at least three times with similar results. **(f)** Comparison of *MYCN* expression in tumours and our engineered hESCs and their derivatives. The plots on the left show bulk RNA-seq data from three NB tumour compendia (CCRI, SEQC, TARGET) divided into cases with and without diagnosed *MYCN* amplification. The plot on the right ("This study") shows our scRNA-seq data divided by cell line (across all timepoints). The values in all plots are the $\log_2$ fold changes over the mean of all non-amplified tumours (left plots) or all WT cells (right plot). **(g)** Immunofluorescence analysis of PERIPHERIN expression in D19 cultures following differentiation of hESCs with the indicated genotypes. Cell nuclei were counterstained using Hoechst 33342. **(h)** Immunofluorescence analysis of PERIPHERIN expression in D28 cultures following differentiation of 17q1q (No DOX) or 17q1qMYCN hESCs following DOX treatment at the indicated timepoints. Cell nuclei were counterstained using Hoechst 33342. For panels g and h all experiments were repeated at least three times with similar results. Source data are provided as a Source Data file. WT, wild-type H7 hESCs; CNA, copy number alteration; SNV, single-nucleotide variant; SEM, standard error of the mean; DOX, Doxycycline; D5/9, day 5/9.

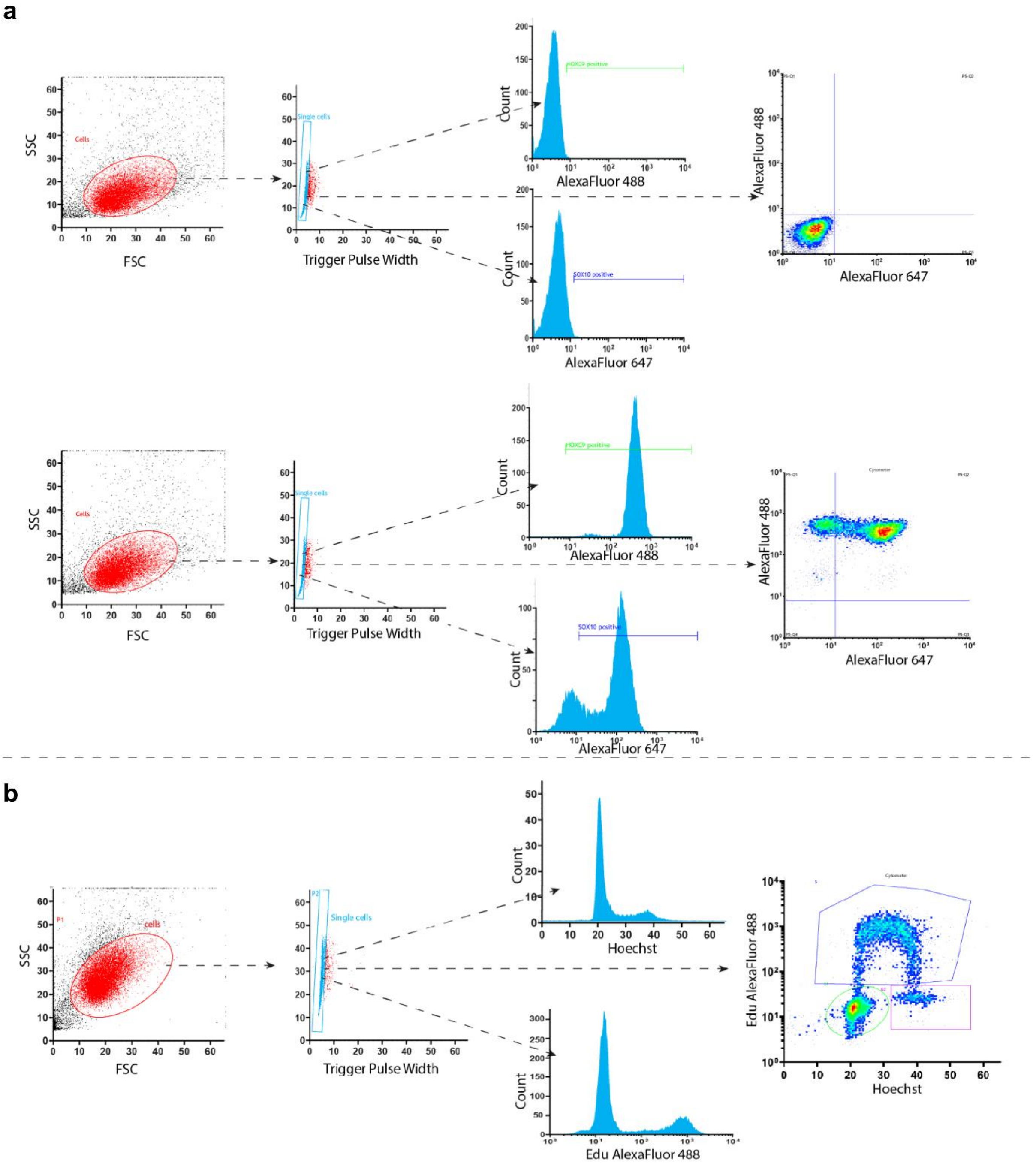

Supplementary Figure 5. Plots illustrating the gating strategy for the FACS performed in this manuscript.

(a) Secondary-only (negative) gating was set using cells differentiated at the corresponding stage stained only with secondary antibodies (see Methods). The scatter plots of Forward Scatter (FSC) and Side scatter (SSC) variables were used to identify the main cell population size. Once the main population was identified, SSC and Trigger Pulse Width variables were further used to determine the single-cell population (doublet discrimination). The FITC (488) and Red (647) channels were used to set the baseline of fluorescence (upper panel) and subsequent positive gates (lower panel). **(b)** Cells were analysed in the flow cytometer (BD FACSJazz) using the 405 nm laser to detect the Hoechst staining and 488 nm to detect the Edu staining. FSC, forward scatter; SSC, side scatter.

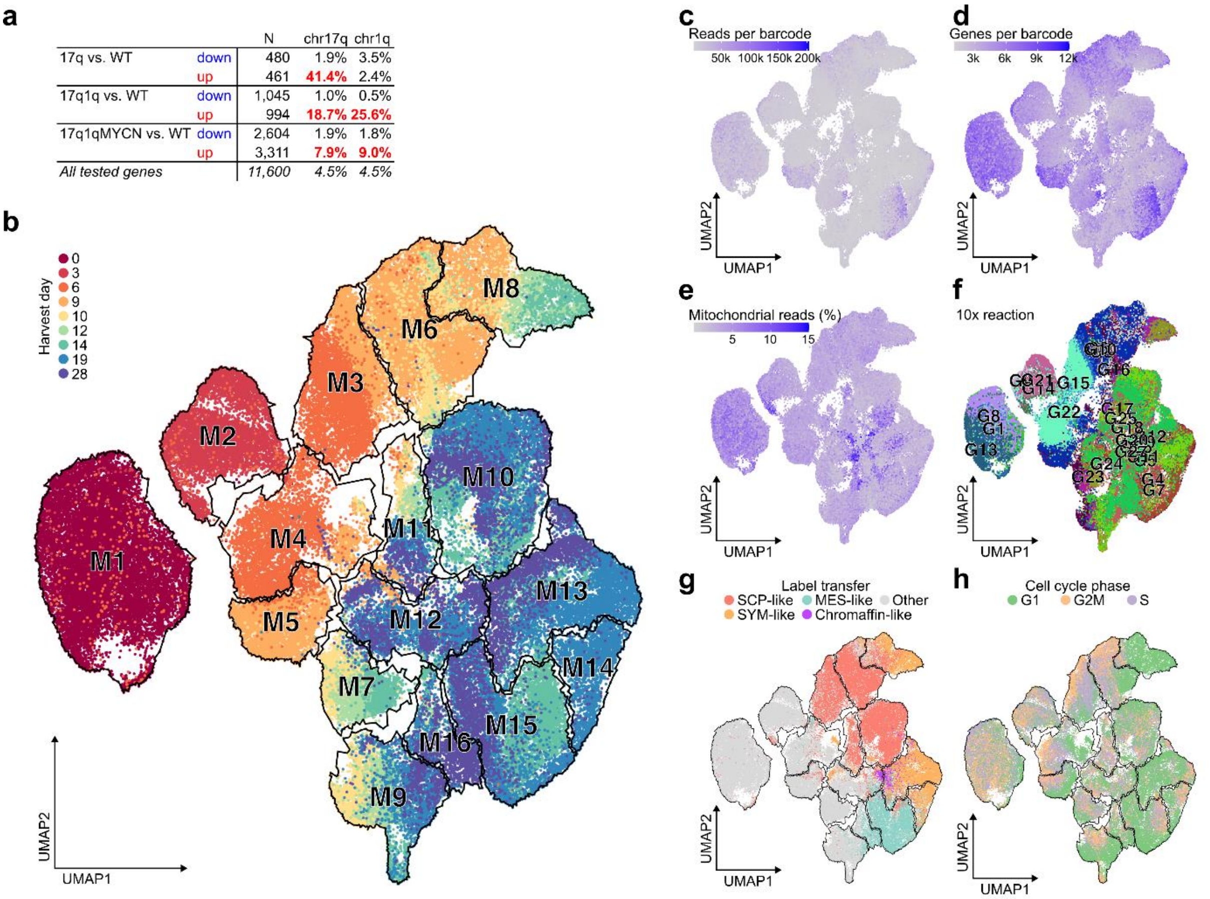

		N	chr17q	chr1q
17q vs. WT	down	480	1.9%	3.5%
	up	461	**41.4%**	2.4%
17q1q vs. WT	down	1,045	1.0%	0.5%
	up	994	**18.7%**	**25.6%**
17q1qMYCN vs. WT	down	2,604	1.9%	1.8%
	up	3,311	**7.9%**	**9.0%**
All tested genes		*11,600*	*4.5%*	*4.5%*

Supplementary Figure 6. scRNA-seq analysis of differentiating wild-type and mutant hESCs.

(a) Overview of the number of DEGs in 17q, 17q1q, and 17q1qMYCN cells at D9 of differentiation compared to WT. The total number of DEGs is given (N), and the percentage of those genes that are located on chromosome arms chr17q or chr1q are indicated. Percentage values >5% have been highlighted (which also correspond to upregulated DEGs within known CNAs). DEGs are reported in Supplementary Data 6. **(b)** Cell clusters defined for the full in-vitro trunk NC dataset generated in this study. Cluster marker genes are reported in Supplementary Data 8. **(c-h)** QC covariate plots for reads per cell (c), features per cell (d), % mitochondrial genes (e), sequencing reaction (f), computationally inferred cell cycle stage (g), and cell cycle phase (h). Source data are provided as a Source Data file. DEG, differentially expressed gene; QC, quality control; WT, wild-type H7 hESCs; UMAP, Uniform Manifold Approximation and Projection; D9, day 9; SCP, Schwann cell precursor; SYM, sympathoblast; MES, mesenchymal.

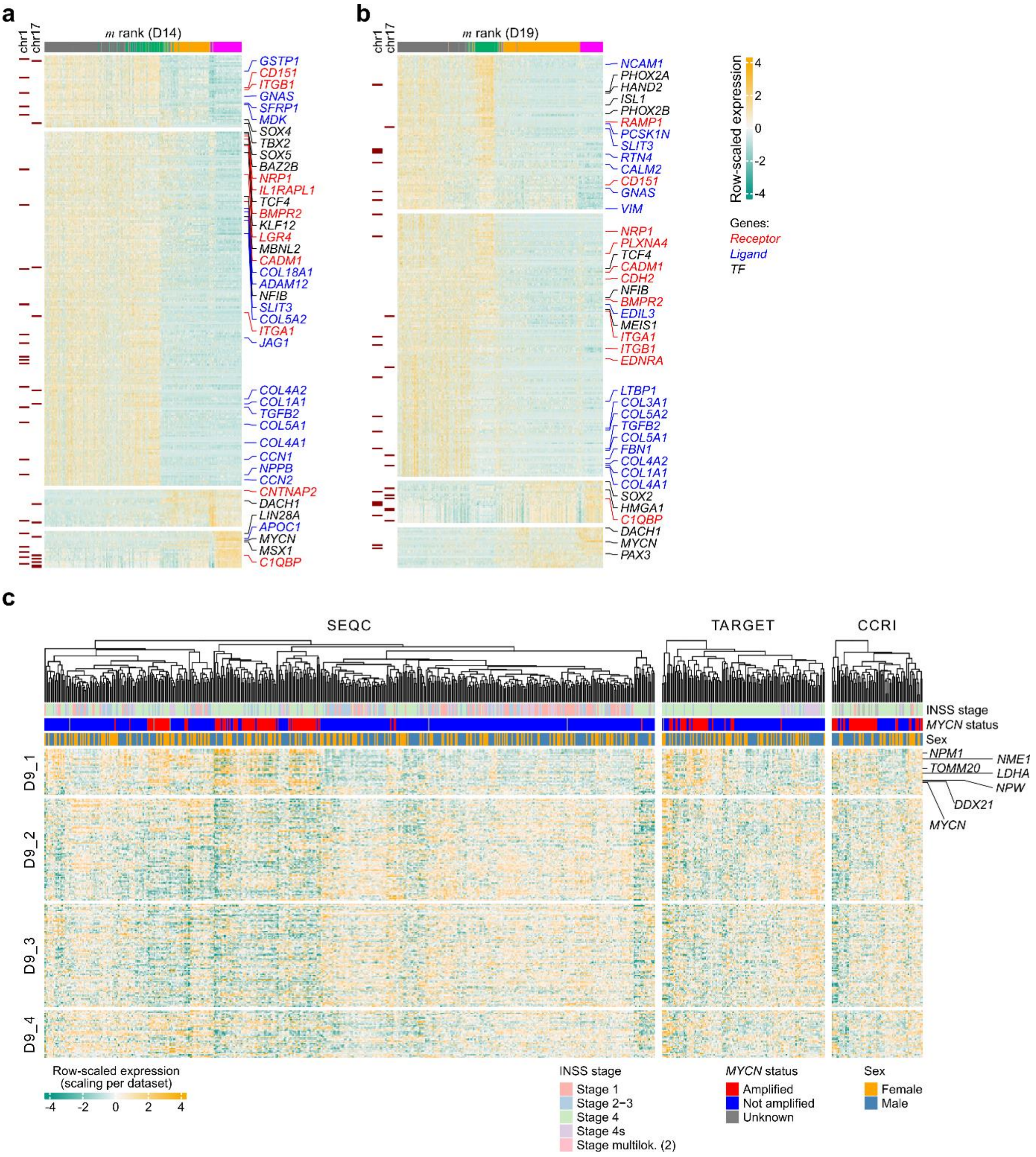

Supplementary Figure 7. Differential gene expression in mutant hESC-derived trunk NC and sympathoadrenal cells.

(a,b) Heatmaps containing the genes correlated or anti-correlated with the mutation score *m* for D14 (**a**) and D19 (**b**). The annotation bars on the left of the heatmaps indicate whether the gene is on chromosome 1q or 17q. Transcription factors (black), receptors (red) and ligands (blue) have been highlighted. All mutation-score-related genes are reported in Supplementary Data 9. **(c)** Heatmap showing the expression of mutation-score-related genes at D9 (cp. Fig. 4c, Supplementary Data 9) in public bulk RNA-seq data from three NB tumour compendia (SEQC, TARGET, CCRI). The heatmaps display the row-normalised transcript counts per gene and sample. The INSS stage, *MYCN* amplification status, and sex of each sample are indicated by the colour bars on top. Genes in D9_1 that are highly expressed in *MYCN*-amplified tumours are highlighted. Source data are provided as a Source Data file. m rank, mutation score rank; NB, neuroblastoma; TF, transcription factor; SEQC, Sequencing Quality

Control project; TARGET, Therapeutically Applicable Research to Generate Effective Treatments project; CCRI, St. Anna Children’s Cancer Research Institute; INSS, International Neuroblastoma Staging System.

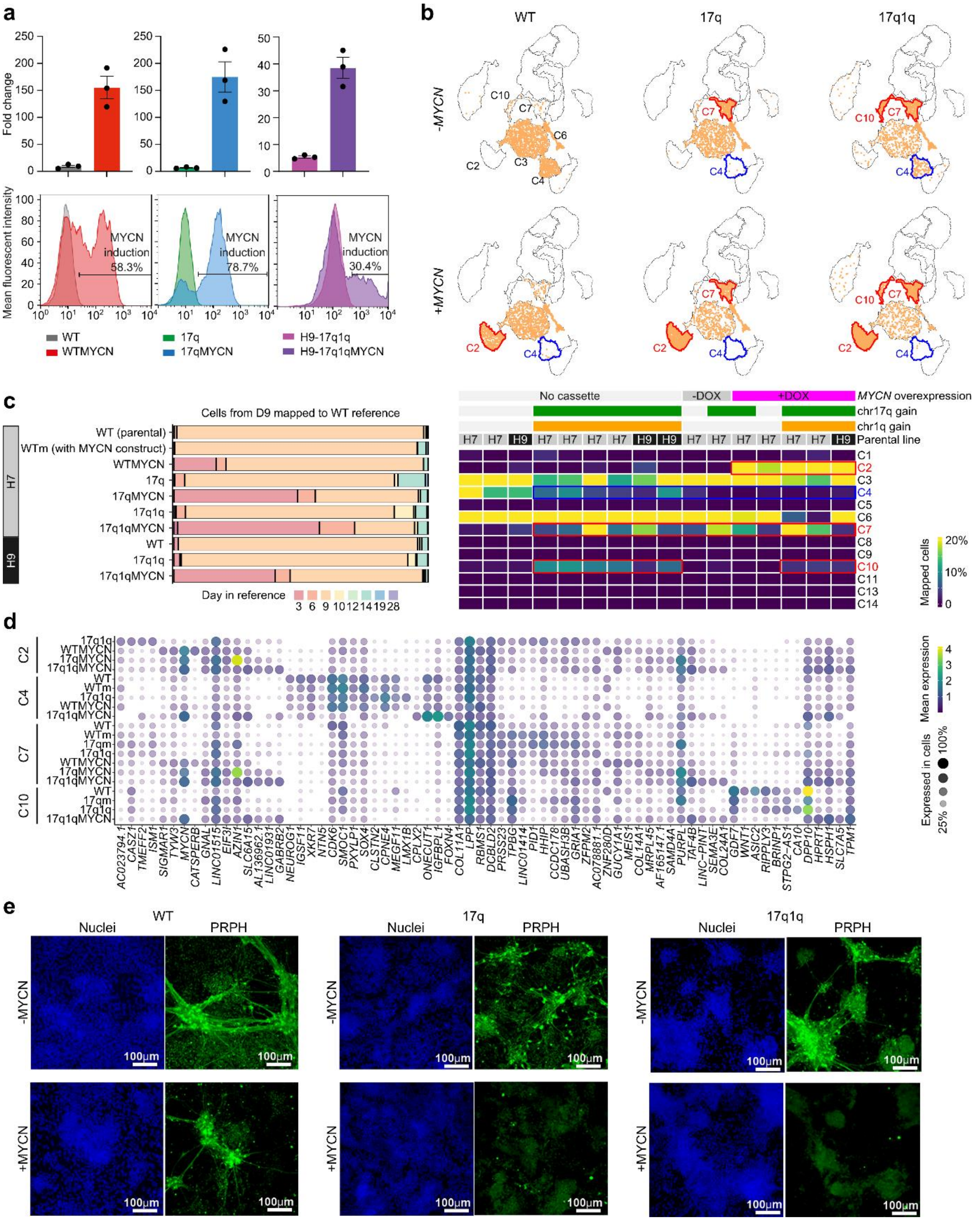

Supplementary Figure 8. Comparison of multiple mutant hESC lines with the WT trunk NC differentiation reference.

(a) Analysis of MYCN expression at the transcript (top) and protein (bottom) level at D9 following differentiation of hESC lines of the indicated genotypes after DOX treatment from D5 vs untreated controls using qPCR and flow cytometry, respectively. Bar plots show the mean of n = 3 biological replicates (error bars = SEM). All flow cytometry experiments were repeated at least three times with similar results. **(b)** scRNA-seq data (Parse Bio) mapped to our WT reference (from Fig. 1). Top: Example glasswork UMAP plots depicting the destination clusters

in the WT reference for WT, 17q, and 17q1q cells (H7 cell line) with and without *MYCN* overexpression. Clusters with an increased (red) and decreased (blue) number of mapped cells are indicated. Bottom: Heatmap displaying the mapping of all 16 datasets including 1-4 replicates of each cell line / experimental condition. Cells were derived from two parental hESC lines (H7 and H9), with/without gains of chr17q and/or chr1q, and edited with a *MYCN* expression cassette (which can be active [+DOX] or inactive [-DOX]). Clusters with increased/decreased numbers of mapped cells have been highlighted with red/blue boxes, respectively. **(c)** Stacked barplots summarising the mappings from panel b for derivatives of each hESC line (top to bottom). Each bar indicates the proportion of cells (at D9) that mapped to cells in the WT reference of a given developmental stage. The plots suggest that cells with *MYCN* induction mapped to earlier stages compared to WT. **(d)** Bubble plot showing marker genes of cells mapped to the WT cell clusters associated with the different genetic changes in panel b (C2, C4, C7 and C10). Only cells with positive percentages are shown. The size and colour of each circle indicate the percent of cells which express the gene and the average expression, respectively. Some gene expression programmes are affected by specific combinations of mutations and cell types, such as polyamine homeostasis gene *AZIN1* or P53 suppressor *PURPL* in 17qMYCN in C2 or C7. **(e)** Immunofluorescence analysis of PERIPHERIN (PRPH) expression at D19 following differentiation of *MYCN*-overexpressing hESCs with the indicated genotypes in the presence and absence of DOX. Cell nuclei were counterstained using Hoechst 33342. All experiments were repeated at least three times with similar results. Source data are provided as a Source Data file. DOX, Doxycycline; WT, wild-type H7 hESCs; SEM, standard error of the mean; D9/19, day 9/19.

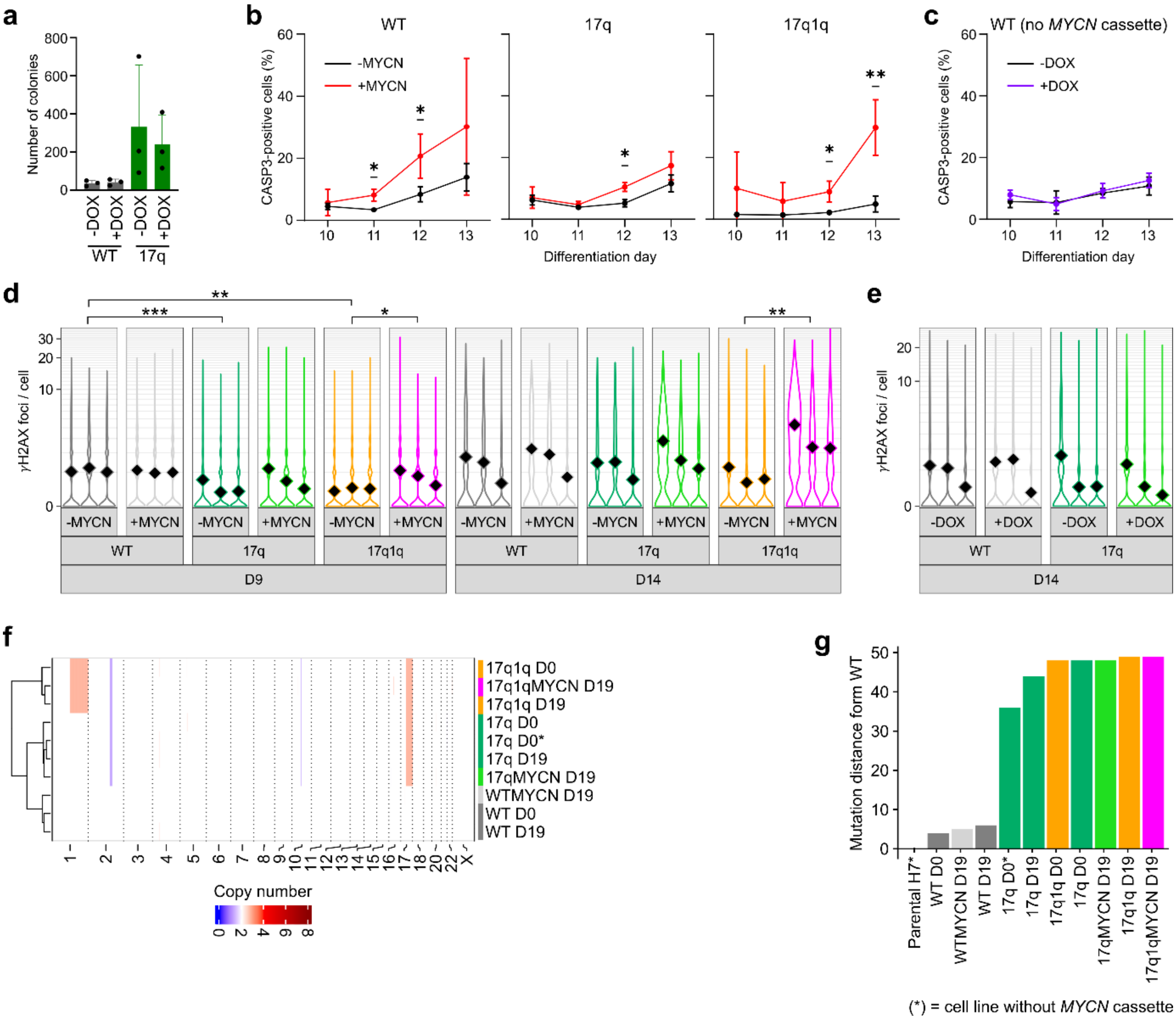

Supplementary Figure 9. Apoptosis, DNA damage, and mutations in mutant hESC-derived trunk NC differentiation.

(a) Comparison of the number of colonies formed by D14 cells from unmodified WT and 17q cells in the presence and absence of DOX treatment with or without 17q gain. Bar plots show the mean of n = 3 biological replicates (error bars = SD). Statistical analysis was performed using ordinary one-way ANOVA with Tukey correction. No statistically significant differences were observed. **(b)** Time course flow cytometry analysis of cleaved Caspase-3 levels in differentiating trunk NC cells generated from hESCs of the indicated genotypes in the presence and absence of DOX treatment from D5. Data points show the mean of at least three biological replicates (error bars = SEM). P-values in comparisons: WTMYCN (D11 DOX vs NO DOX, p = 0.010797 = *; D12 DOX vs NO DOX, p = 0.033974 = *), 17qMYCN (D12 DOX vs NO DOX, p = 0.034166 = *), 17q1qMYCN (D12 DOX vs NO DOX, p = 0.020679 = *; D13 DOX vs NO DOX, p = 0.005686 = **). **(c)** Control comparison of Caspase-3 levels in untreated and DOX-treated WT controls. As in panel b. **(d)** Number of γH2AX foci per cell in differentiating cultures corresponding to indicated timepoints and genotypes in the presence and absence of DOX treatment. The diamond symbols represent the mean. FDR values in comparisons (ANOVA with mixed effect model with negative binomial distribution): FDR *** < 0.001, ** < 0.01, * < 0.05. Comparisons performed: DOX status within a genotype and day, genotype vs WT per DOX status and day. **(e)** Control comparison of the number of γH2AX foci per cell in untreated and DOX-treated unmodified WT and 17q cells. As in panel d. **(f)** Heatmap containing CNA calls by Sequenza[6] in bins of 1Mbp. Dendrogram represents hierarchical clustering. CNA coordinates are reported in Supplementary Data 5. **(g)**

Phylogenetic distance measured in number of mutations of each sample relative to the parental control using the phylogenetic analysis from Figure 5e. Colours in panels d-f have been chosen consistently to represent the different cell lines / experimental conditions. Source data are provided as a Source Data file. WT, wild-type H7 hESCs; D5/11/12/13/14, day 5/11/12/13/14; DOX, Doxycycline; ANOVA, analysis of variance; SD, standard deviation, FDR, false discovery rate; CNA, copy number alteration.

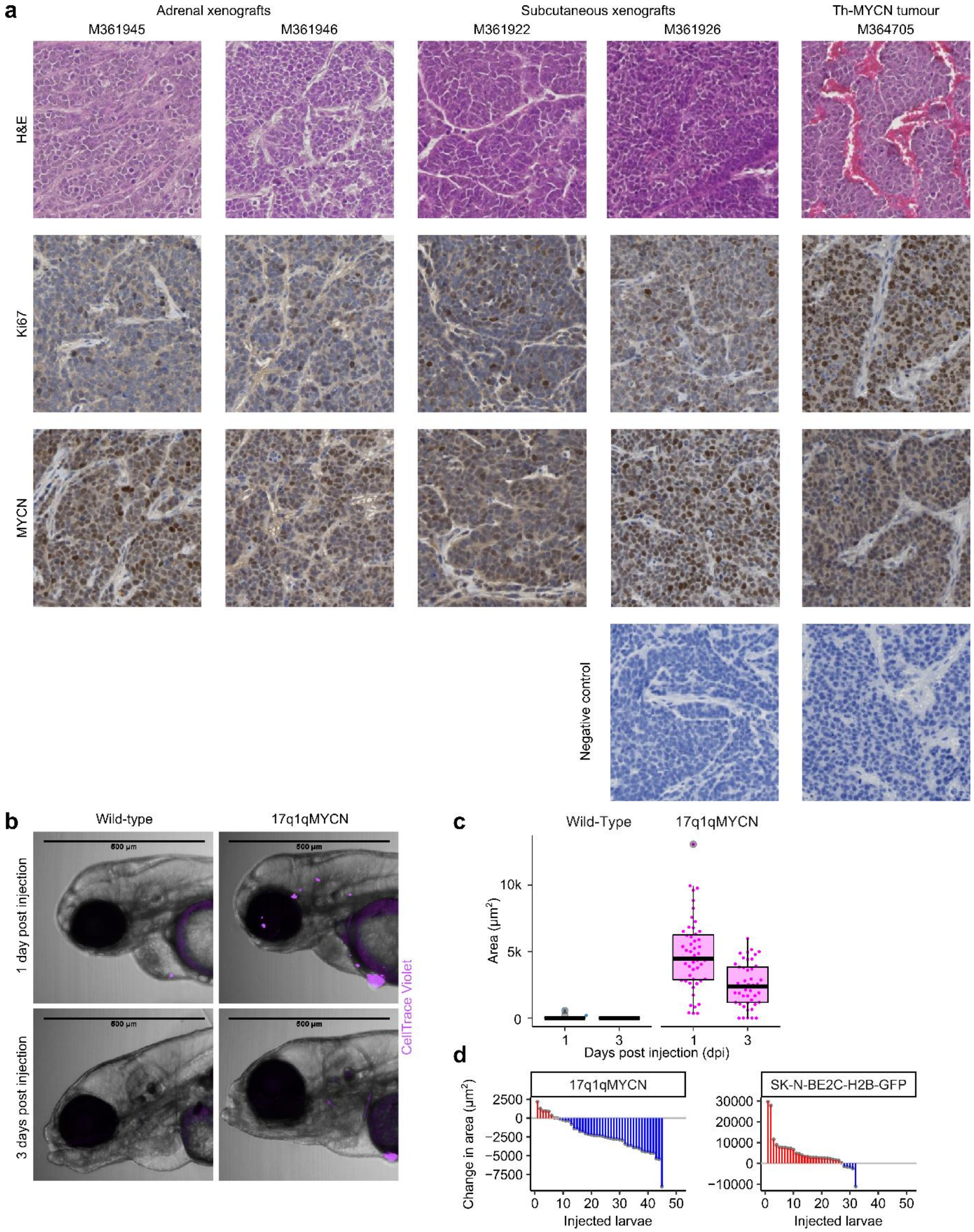

Supplementary Figure 10. Mouse and zebrafish xenografts of WT, 17q1q, and 17q1qMYCN cells.

(a) Representative sections from individual tumours following xenografting of 17q1qMYCN cells in the indicated locations and showing haematoxylin and eosin staining (top row), Ki67 (middle row), and MYCN expression (bottom row). Tumour sections from a Th-MYCN NB GEM model and negative controls are shown. **(b)** Representative images of zebrafish xenografted with WT or 17q1qMYCN cells labelled with CellTrace Violet at one day or three days post injection (top and bottom, respectively). **(c)** Quantification of the area covered by

WT or 17q1qMYCN cells in zebrafish xenografts at 1dpi and 3dpi. The lower/upper hinges of the boxplots report the 25th/75th percentile, and the whiskers extend to 1.5 times the inter-quartile range. The median is indicated by a horizontal line. While some 17q1qMYCN cells persisted, WT cells did not survive in zebrafish xenografts. Xenografts with WT cells (n = 11), and 17q1qMYCN cells (n = 51). **(d)** Waterfall plots depicting the change in tumour area for 17q1qMYCN at D9 of differentiation (~ trunk neural crest stage) and SK-N-BE2C-H2B-GFP[8] cells in zebrafish xenografts from 1dpi to 3dpi. H&E, haematoxylin and eosin; NB, neuroblastoma; GEM, genetically engineered mouse; D9, day 9.

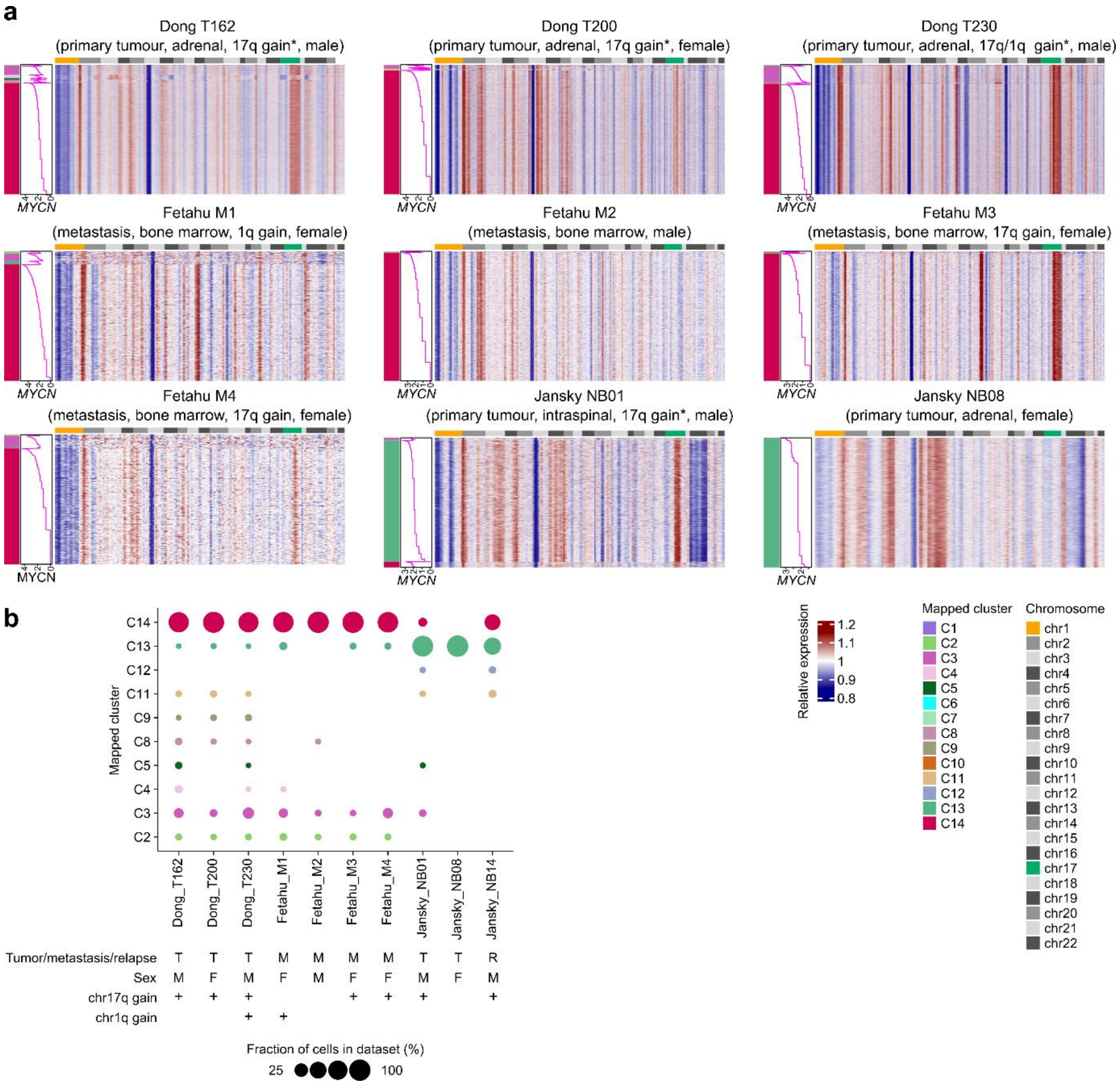

Supplementary Figure 11. Analysis of *MYCN*-amplified tumour cells and mapping to in-vitro trunk NC differentiation.

(a) inferCNV[9] profile heatmaps such as the one in Fig. 7b for the remaining 9 tumour datasets[2,10,11] not shown in Fig. 7. Each row (tumour cells) and each column (genes, ordered by genomic position), indicate the intensity of the CNA signal relative to non-tumour, haematopoietic cells from the same sample. All samples were curated and processed as described in Fig. 6 and mapped to our wild-type reference (cp Fig. 1). Cells are ordered first by matching cluster and then by *MYCN* levels within each cluster. Annotation of the chromosomes can be found on top of the heatmap. The tumour type (primary, metastasis, relapse), tissue, chr17q/1q gain status, and sex of each sample are indicated. The asterisk (*) marks cases in which the chr17q/1q gain status is based on our inspection of the inferred inferCNV copy number profile shown in this figure panel. (**b)** Bubble plot summarising the mapping of tumour cells to our wild-type reference. The tumour type (primary, metastasis, relapse), sex, and chr17q/1q gain status are indicated. Ordering by study suggests a study-specific effect; for example, samples from Fetahu et al. are all bone marrow metastases and lack mesenchymal C11 cells. CNA, copy number alteration; T, primary tumour; M, metastasis; R, relapse; M, male; F, female

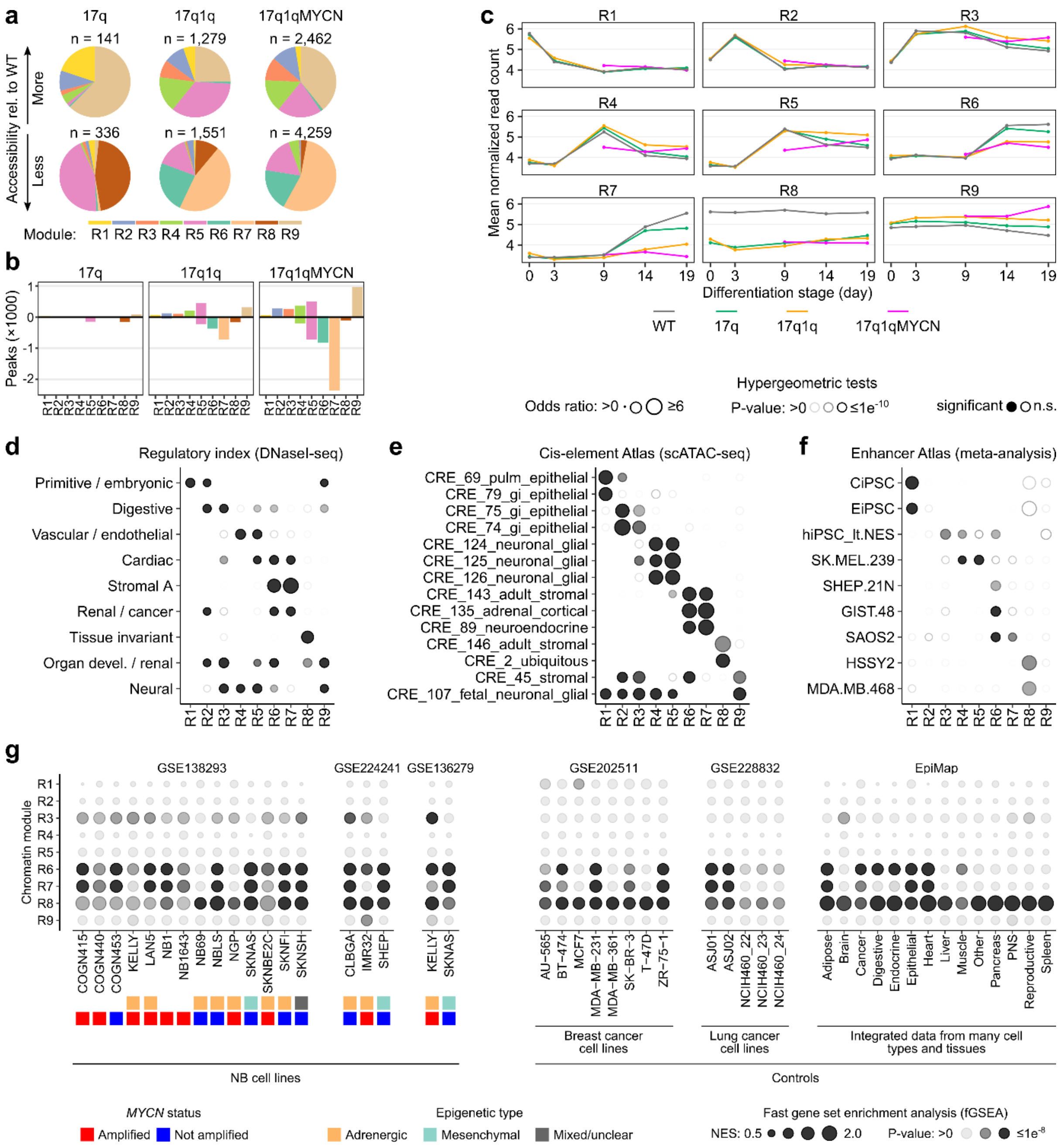

Supplementary Figure 12. Chromatin accessibility in differentiating wild-type and mutant hESCs.

(a) Pie charts indicating the fraction of differentially accessible regions belonging to chromatin modules (Fig. 9a) in mutant hESCs compared to WT (left to right). Up- and down-regulated regions are shown separately (top vs. bottom row). The total number (*n*) of regions in each category is indicated. **(b)** Barplots indicating the number of up- (positive numbers) and down-regulated (negative numbers) peaks from each comparison of mutant hESCs vs WT, split by chromatin module (R1-R9). **(c)** Line plots summarizing the dynamics of accessibility per module and cell line throughout differentiation. Each data point represents the mean normalised read count for all regions belonging to the indicated module across all replicates of the indicated cell line (different colours). **(d-f)** Enrichment analysis of overlaps between regions belonging to the nine chromatin modules (from left to right) and annotated reference regions from the Regulatory Index[12] (based on DNaseI-seq; d), Cis-element Atlas[13] (based on scATAC-seq analysis; e) and the Enhancer Atlas[14] (based on a meta-analysis of many different data; f). The size and transparency of circles indicate the odds ratio and P-value,

respectively (hypergeometric test, hypeR[15]). Significant results are indicated with filled circles ($P_{adj} \leq 0.05$; P-values have been adjusted for multiple hypothesis testing using the Benjamini-Hochberg method). The top enrichments per stage have been selected for visualisation and all results including exact P-values are reported in Supplementary Data 14. **(g)** Bubble plots showing the outputs of a fast gene set enrichment analysis (*fgsea*[16]) of open chromatin in external data compared to our chromatin modules. Each sub-panel indicates data from a different source (from left to right) including three collections of NB cell lines[17–19] (GSE138293, GSE224241, GSE136279), two adult cancer cell lines [20,21] (GSE202511, GSE228832), and a meta-analysis of human tissue data[22] (https://epigenome.wustl.edu/epimap). The epigenetic type[23,24] and *MYCN* amplification status of each NB cell line are indicated. Source data are provided as a Source Data file. NB, neuroblastoma; WT, wild-type H7 hESCs; R1-R9, chromatin modules identified in Fig. 9a; NES, normalised enrichment score; n.s., not significant.

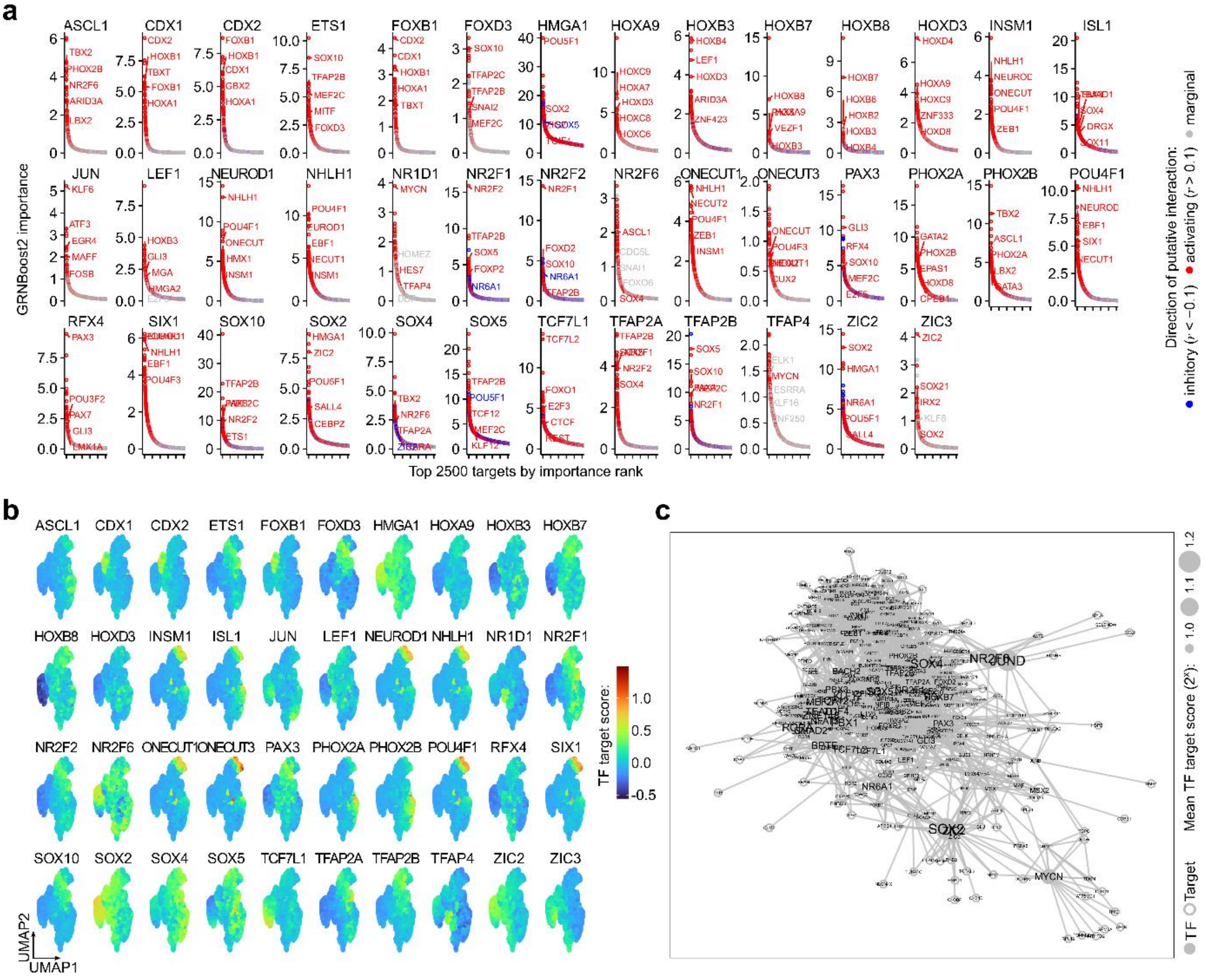

Supplementary Figure 13. Analysis of transcription factor target sets and gene-regulatory networks.

(a) Top-2500 targets of selected TFs as predicted by GRNboost2 algorithm[25] based on our scRNA-seq data. Putative targets without support in our ATAC-seq data (motif for TF in ≥1 peak near the gene) have been removed. We also calculated the Pearson correlation coefficient (*r*) between each TF and target gene to determine the direction of the putative interaction (*r* > 0.1, “activating”; *r* < -0.1, “inhibitory”; others, “marginal”). The top TFs in the target lists have been highlighted. TF target gene sets are reported in Supplementary Data 15. (**b)** Average expression (Seurat module score) of the target gene sets (matching “activating” targets of the TFs in panel a in our integrated scRNA-seq dataset (cp. Fig. 4a). (**c)** Gene-regulatory networks diagram visualising putative TF to target interactions for the genes in gene sets D9_1 to D9_4 (cp. Fig. 4b,c) and enriched TF targets (cp. Fig. 10d) In these diagrams, each node represents a TF or target gene, and each edge is a link between a TF and a target. Node size is proportional to the mean target score of the indicated TFs (fixed size for non-TF nodes). Source data are provided as a Source Data file. TF, transcription factor; r, Pearson correlation coefficient.

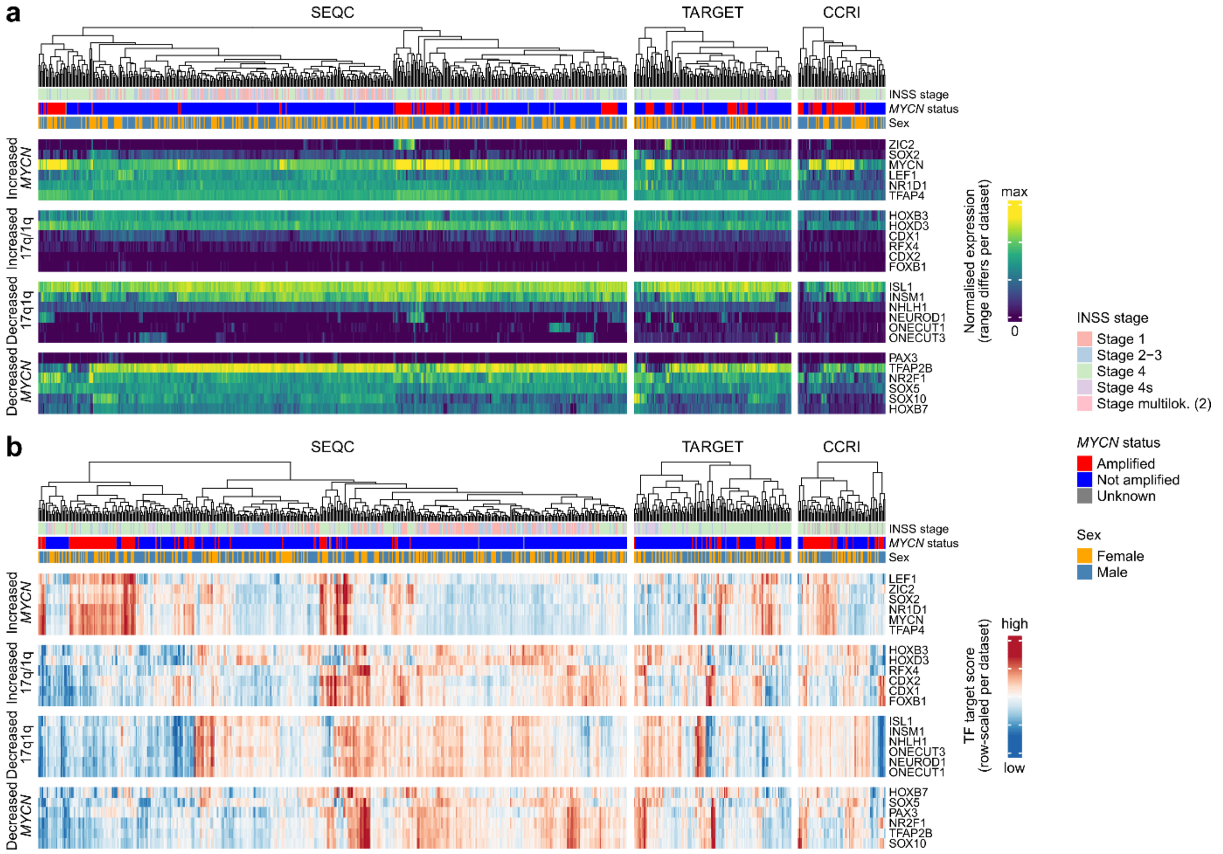

Supplementary Figure 14. Expression of transcription factors and their target genes in public RNA-seq datasets.

(a) Expression of 24 selected TFs (from Fig. 10e) in public bulk RNA-seq data from three NB tumour compendia (SEQC, TARGET, CCRI). The heatmaps display the normalised transcript counts per gene and sample, colours have been scaled from 0 to the maximum per source dataset (dark blue to yellow). The INSS stage, *MYCN* amplification status, and sex of each sample are indicated by the colour bars on top. Some of the examined TFs are not or very weakly expressed in the investigated samples from tumours. (**b)** Heatmaps for the same data and TFs as in panel a, but here heatmap values indicate the TF target score (Seurat module score), a summary of the expression of putative target genes of each TF in the respective sample. TFs with high activity in 17q1qMYCN cells (the 6 TFs on top) are active in *MYCN*-amplified samples. Source data are provided as a Source Data file. NB, neuroblastoma; SEQC, Sequencing Quality Control project; TARGET, Therapeutically Applicable Research to Generate Effective Treatments project; CCRI, St. Anna Children's Cancer Research Institute; INSS, International Neuroblastoma Staging System; TF, transcription factor.

SUPPLEMENTARY REFERENCES

1. Kameneva, P. *et al.* Single-cell transcriptomics of human embryos identifies multiple sympathoblast lineages with potential implications for neuroblastoma origin. *Nat Genet* **53**, 694–706 (2021).

2. Jansky, S. *et al.* Single-cell transcriptomic analyses provide insights into the developmental origins of neuroblastoma. *Nat Genet* **53**, 683–693 (2021).

3. Street, K. *et al.* Slingshot: Cell lineage and pseudotime inference for single-cell transcriptomics. *BMC Genomics* **19**, 477 (2018).

4. van den Berge, K. *et al.* Trajectory-based differential expression analysis for single-cell sequencing data. *Nat Commun* **11**, 1–13 (2020).

5. La Manno, G. *et al.* RNA velocity of single cells. *Nature* **560**, 494–498 (2018).

6. Favero, F. *et al.* Sequenza: allele-specific copy number and mutation profiles from tumor sequencing data. *Ann Oncol* **26**, 64–70 (2015).

7. Abbasi, M. R. *et al.* Impact of Disseminated Neuroblastoma Cells on the Identification of the Relapse-Seeding Clone. *Clin Cancer Res* **23**, 4224–4232 (2017).

8. Wrobel, J. K. *et al.* Rapid In Vivo Validation of HDAC Inhibitor-Based Treatments in Neuroblastoma Zebrafish Xenografts. *Pharmaceuticals (Basel)* **13**, 345 (2020).

9. Patel, A. P. *et al.* Single-cell RNA-seq highlights intratumoral heterogeneity in primary glioblastoma. *Science (1979)* **344**, 1396–1401 (2014).

10. Dong, R. *et al.* Single-Cell Characterization of Malignant Phenotypes and Developmental Trajectories of Adrenal Neuroblastoma. *Cancer Cell* **38**, 716-733.e6 (2020).

11. Fetahu, I. S. *et al.* Single-cell transcriptomics and epigenomics unravel the role of monocytes in neuroblastoma bone marrow metastasis. *Nat Commun* **14**, 3620 (2023).

12. Meuleman, W. *et al.* Index and biological spectrum of human DNase I hypersensitive sites. *Nature 2020 584:7820* **584**, 244–251 (2020).

13. Zhang, K. *et al.* A single-cell atlas of chromatin accessibility in the human genome. *Cell* **184**, 5985-6001.e19 (2021).

14. Gao, T. & Qian, J. EnhancerAtlas 2.0: an updated resource with enhancer annotation in 586 tissue/cell types across nine species. *Nucleic Acids Res* **48**, D58–D64 (2020).

15. Federico, A. & Monti, S. hypeR: an R package for geneset enrichment workflows. *Bioinformatics* **36**, 1307–1308 (2020).

16. Korotkevich, G. *et al.* Fast gene set enrichment analysis. *bioRxiv* 060012 (2021) doi:10.1101/060012.

17. Upton, K. *et al.* Epigenomic profiling of neuroblastoma cell lines. *Sci Data* **7**, (2020).

18. Decaesteker, B. *et al.* SOX11 regulates SWI/SNF complex components as member of the adrenergic neuroblastoma core regulatory circuitry. *Nature Communications 2023 14:1* **14**, 1–16 (2023).

19. Gartlgruber, M. *et al.* Super enhancers define regulatory subtypes and cell identity in neuroblastoma. *Nat Cancer* **2**, 114–128 (2021).

20. Xu, D. *et al.* Recapitulation of patient-specific 3D chromatin conformation using machine learning. *Cell reports methods* **3**, (2023).

21. Hariprakash, J. M. *et al.* Leveraging Tissue-Specific Enhancer-Target Gene Regulatory Networks Identifies Enhancer Somatic Mutations That Functionally Impact Lung Cancer. *Cancer Res* **84**, (2024).

22. Boix, C. A., James, B. T., Park, Y. P., Meuleman, W. & Kellis, M. Regulatory genomic circuitry of human disease loci by integrative epigenomics. *Nature 2021 590:7845* **590**, 300–307 (2021).

23. Boeva, V. *et al.* Heterogeneity of neuroblastoma cell identity defined by transcriptional circuitries. *Nat Genet* **49**, 1408–1413 (2017).

24. Van Groningen, T. *et al.* Neuroblastoma is composed of two super-enhancer-associated differentiation states. *Nat Genet* **49**, 1261–1266 (2017).

25. Moerman, T. *et al.* GRNBoost2 and Arboreto: Efficient and scalable inference of gene regulatory networks. *Bioinformatics* **35**, 2159–2161 (2019).
