# Supplementary material for: A human neural crest model reveals the developmental impact of neuroblastoma-associated chromosomal aberrations
Source: Nat Commun. 2024 May 3;15:3745. doi: 10.1038/s41467-024-47945-7 (PMC11068915; doi:10.1038/s41467-024-47945-7)
Supplement: Supplementary file 4 — Description of Additional Supplementary Files [file 41467_2024_47945_MOESM4_ESM.pdf]

Description of Additional Supplementary Files

Supplementary Data 1: scRNA-seq, WES, and ATAC-seq dataset overview

Supplementary Data 2: scRNA-seq cluster marker genes (WT)

Supplementary Data 3: scRNA-seq markers of SCP-SYM-MES transition states

Supplementary Data 4: Whole-exome sequencing SNVs.

Supplementary Data 5: Whole-exome sequencing CNAs.

Supplementary Data 6: scRNA-seq MUT vs. WT differentially expressed genes.

Supplementary Data 7: scRNA-seq MUT vs. WT enrichment results

Supplementary Data 8: scRNA-seq cluster marker genes (WT+MUT)

Supplementary Data 9: scRNA-seq genes correlated to mutations.

Supplementary Data 10: Tumour-in vitro markers

Supplementary Data 11: Survival analyses

Supplementary Data 12: ATAC-seq regions (peaks) and chromatin modules

Supplementary Data 13: ATAC-seq differential accessibility analysis

Supplementary Data 14: ATAC-seq chromatin module enrichment results

Supplementary Data 15: Transcription factor target genes

Supplementary Data 16: Transcription factor enrichments
