# Supplementary material for: A human neural crest model reveals the developmental impact of neuroblastoma-associated chromosomal aberrations
Source: Nat Commun. 2024 May 3;15:3745. doi: 10.1038/s41467-024-47945-7 (PMC11068915; doi:10.1038/s41467-024-47945-7)
Supplement: Supplementary file 21 — Reporting summary [file 41467_2024_47945_MOESM21_ESM.pdf]

nature portfolio

Corresponding author(s): Florian Halbritter & Anestis Tsakiridis

Last updated by author(s): Mar 18, 2024

Reporting Summary

Nature Portfolio wishes to improve the reproducibility of the work that we publish. This form provides structure for consistency and transparency in reporting. For further information on Nature Portfolio policies, see our Editorial Policies and the Editorial Policy Checklist.

Statistics

For all statistical analyses, confirm that the following items are present in the figure legend, table legend, main text, or Methods section.

n/a	Confirmed	
☐	☒	The exact sample size (*n*) for each experimental group/condition, given as a discrete number and unit of measurement
☐	☒	A statement on whether measurements were taken from distinct samples or whether the same sample was measured repeatedly
☐	☒	The statistical test(s) used AND whether they are one- or two-sided<br>*Only common tests should be described solely by name; describe more complex techniques in the Methods section.*
☐	☒	A description of all covariates tested
☒	☐	A description of any assumptions or corrections, such as tests of normality and adjustment for multiple comparisons
☐	☒	A full description of the statistical parameters including central tendency (e.g. means) or other basic estimates (e.g. regression coefficient) AND variation (e.g. standard deviation) or associated estimates of uncertainty (e.g. confidence intervals)
☐	☒	For null hypothesis testing, the test statistic (e.g. *F*, *t*, *r*) with confidence intervals, effect sizes, degrees of freedom and *P* value noted<br>*Give P values as exact values whenever suitable.*
☒	☐	For Bayesian analysis, information on the choice of priors and Markov chain Monte Carlo settings
☒	☐	For hierarchical and complex designs, identification of the appropriate level for tests and full reporting of outcomes
☐	☒	Estimates of effect sizes (e.g. Cohen's *d*, Pearson's *r*), indicating how they were calculated

Our web collection on statistics for biologists contains articles on many of the points above.

Software and code

Policy information about availability of computer code

Data collection	Flow cytometry data: BD FACS Software 1.2.0.142, CytExpert<br>Immunofluorescence images: InCell Analyzer 2200<br>Time-lapse microscopy: Nikon Biostation CT<br>qPCR: QuantStudio 12K Flex Real-Time PCR Software<br>MRI: Horos<br>Apart from mentioned software, only on-board instrument and no other special software was used for data collection.

Data analysis

Image processing: ImageJ 1.52f, CellProfiler 4.2.1, PerkinElmer Harmony v4.9
Whole-exome sequencing: nfcore sarek v2.7.2, bcftools v1.9, VEP v99.2, Sequenza v3.0.0, VARAN-GIE v0.2.9, ggplot v3.3.5, rtracklayer v1.54.0
scRNA-seq (10x): CellRanger 7.1.0, R v4.1.3, SCTransform v0.3.3, EmptyDrops v1.14.2, scDblFinder v1.8.0, hypeR v1.10.0, edgeR v3.32.1, Slingshot v2.2.0, inferCNV v1.10.1, GSVA v1.42.0, survival v3.3-1, DESeq2 v1.34.0, Seurat v4.1.0, DElegate v1.1.0, sceasy v0.0.7, reticulate v1.24, scvi v0.20.3, Python 3.11
scRNA-seq (Parse): split-pipe v1.0.6p, R v4.1.3, Seurat v4.1.0, canceRbits v0.16.0, DElegate v1.1.0
ATAC-seq: PEPATAC v0.9.5, R v4.1.3, ggplot 3.3.5, ComplexHeatmap v2.10.0, Rsubread v2.8.2, DESeq2 v1.34.0, canceRbits v0.1.6, hypeR v1.10.0, motfmatchr v1.16.0, liftOver v1.18.0, GenomicRanges v1.46.1, plyranges v1.14.0, fgsea v1.20.0, Python 3.8.17, arboreto v0.1.6, changepoint v2.2.3, igraph v1.3.1, reticulate v1.24
Flow cytometry data analysis: FlowJo v 10.8.1
Plotting and statistical analysis: GraphPad Prism v9, SAS 9.4
Figure design, post-processing: Inkscape 1.3.2
No custom software was generated in this study. All scripts used for data analysis are available via GitHub https://github.com/cancerbits/saldana_montano2024_ncnb

For manuscripts utilizing custom algorithms or software that are central to the research but not yet described in published literature, software must be made available to editors and reviewers. We strongly encourage code deposition in a community repository (e.g. GitHub). See the Nature Portfolio guidelines for submitting code & software for further information.

Data

Policy information about availability of data

All manuscripts must include a data availability statement. This statement should provide the following information, where applicable:

- Accession codes, unique identifiers, or web links for publicly available datasets
- A description of any restrictions on data availability
- For clinical datasets or third party data, please ensure that the statement adheres to our policy

The single-cell RNA-seq and ATAC-seq data generated in this study have been deposited in the Gene Expression Omnibus (GEO) under accession code GSE219153 [https://www.ncbi.nlm.nih.gov/geo/query/acc.cgi?acc=GSE219153]. The public scRNA-seq data from NB tumours used in this study are available in GEO under the accession codes GSE147821 [https://www.ncbi.nlm.nih.gov/geo/query/acc.cgi?acc=GSE147821], GSE216176 [https://www.ncbi.nlm.nih.gov/geo/query/acc.cgi?acc=GSE216176], and GSE137804 [https://www.ncbi.nlm.nih.gov/geo/query/acc.cgi?acc=GSE137804], and in the European Genome-Phenome Archive (EGA) under accession code EGAS00001004388 [https://ega-archive.org/studies/EGAS00001004388]. Public ATAC-seq data from NB cell lines and controls used in this study are available in GEO under accession codes GSE138293 [https://www.ncbi.nlm.nih.gov/geo/query/acc.cgi?acc=GSE138293], GSE224241 [https://www.ncbi.nlm.nih.gov/geo/query/acc.cgi?acc=GSE224241], GSE136279 [https://www.ncbi.nlm.nih.gov/geo/query/acc.cgi?acc=GSE136279], GSE202511 [https://www.ncbi.nlm.nih.gov/geo/query/acc.cgi?acc=GSE202511], and GSE228832 [https://www.ncbi.nlm.nih.gov/geo/query/acc.cgi?acc=GSE228832], and from the EpiMap website (https://epigenome.wustl.edu/epimap). Bulk RNA-seq data from NB tumours are available in GEO under accession codes GSE49711 [https://www.ncbi.nlm.nih.gov/geo/query/acc.cgi?acc=GSE49711], GSE94035 [https://www.ncbi.nlm.nih.gov/geo/query/acc.cgi?acc=GSE94035], GSE147635 [https://www.ncbi.nlm.nih.gov/geo/query/acc.cgi?acc=GSE147635], and GSE172184 [https://www.ncbi.nlm.nih.gov/geo/query/acc.cgi?acc=GSE172184], and in dbGAP under accession code phs000467 [https://www.ncbi.nlm.nih.gov/projects/gap/cgi-bin/study.cgi?study_id=phs000218]. Source data are provided with this paper. Additionally, processed data from this paper can be accessed and browsed interactively via our GitHub page [https://github.com/cancerbits/saldana_montano2024_ncnb/] and via the R2 Genomics Analysis and Visualization Platform [http://r2platform.com/halbritter24/].

Research involving human participants, their data, or biological material

Policy information about studies with human participants or human data. See also policy information about sex, gender (identity/presentation), and sexual orientation and race, ethnicity and racism.

Reporting on sex and gender

The hESC lines used in this paper have been derived from female donors.

Genomics data from human research participants (neuroblastoma tumor single-cell and bulk RNA-seq data) were obtained from databases on/or the corresponding authors. The sex of donors has been indicated in corresponding figures.

Sex was not included in the parameters for the data analysis results presented. This information is stated in the manuscript and/or methods.

Reporting on race, ethnicity, or other socially relevant groupings

Neither race, ethnicity, nor other socially relevant groupings other than sex/gender were recorded in our study.

Population characteristics

Population characteristic of a human tumour datasets used in this study are described in the original publications. Briefly, we collected scRNA-seq data for tumours with reported MYCN amplification from three sources:
- Three samples (all primary adrenal, 2 male [Dong_T162, Dong_T230], 1 female [Dong_T200]; accession GSE137804 [Gene Expression Omnibus]),
- three samples (2 primary adrenal , 1 relapse/occipital subcutaneous bone metastasis [Jansky_NB14]; 1 female [Jansky_NB08], 2 male [Jansky_NB01, Jansky_NB14]; accession EGAS00001004388 [European Genome-Phenome Archive]),
- and four samples (all metastatic bone marrow; 3 female [Fetahu_M1, Fetahu_M3, Fetahu_M4], 1 male [Fetahu_M2]; accession GSE216176 [Gene Expression Omnibus]).

Furthermore, data from three collections of neuroblastoma RNA-seq data were used, comprising data from NB patients from a diverse range of sexes and other population characteristics (metadata available from the respective sources): phs000467 (Genomic Data Commons), GSE49711, GSE94035, GSE147635 and GSE172184 (Gene Expression Omnibus).

Recruitment	No donor recruitment was performed for this study.
Ethics oversight	Use of hES cells has been approved by the Human Embryonic Stem Cell UK Steering Committee (SCSC23-29).<br><br>This study did not generate any new genomics data from patients. However, we performed re-analyses of previously published (sc)RNA-seq and SNP-array data that was previously collected at our institutions. The collection and research use of human tumour specimen was performed according to the guidelines of the Council for International Organizations of Medical Sciences (CIOMS) and World Health Organisation (WHO) and has been approved by the ethics board of the Medical University of Vienna (Ethikkommission Medizinische Universität Wien; EK2281/2016, 1216/2018, 1754/2022). Informed consent has been obtained from all patients or parents/guardians/legally authorized representatives. The age-adapted informed consent for the CCRI Biobank covers the use of left over materials from medically necessary surgery or biopsy, which after completion of routine diagnostic procedures is biobanked (EK1853/2016) and available for research purposes, including genetic analysis, that are further specified in EK1216/2018 and EK1754/2022: to conduct genetic and transcriptomic analysis and link to clinical data for survival analysis. All data obtained from external sources derived from studies where informed consent was given for broad research use.

Note that full information on the approval of the study protocol must also be provided in the manuscript.

Field-specific reporting

Please select the one below that is the best fit for your research. If you are not sure, read the appropriate sections before making your selection.

☒ Life sciences ☐ Behavioural & social sciences ☐ Ecological, evolutionary & environmental sciences

For a reference copy of the document with all sections, see nature.com/documents/nr-reporting-summary-flat.pdf

Life sciences study design

All studies must disclose on these points even when the disclosure is negative.

Sample size	No statistical sample size calculations were performed. We carried out 2-7 independent sets of experiments for all genomics data generated in this study (scRNA-seq, RNA-seq, ATAC-seq), which is similar to other studies in the field (e.g., https://doi.org/10.1038/s41586-023-06984-8, https://doi.org/10.1038/s41467-022-30557-4).
Data exclusions	scRNA-seq: For one 10X Genomics dataset (G2_GEX), we failed to generate cDNA libraries and this was excluded from the study.<br><br>For all remaining 10X datasets, we performed QC per dataset. We first removed cells with less than 1000 RNA counts and mitochondrial read percentage above 15%. We then used clusters devoid of markers or characterised by markedly higher mitochondrial expression, to derive a library-specific UMI count threshold to further remove low-quality or empty cells. Cutoffs for mRNA counts per cell are in the range 3.4-4 (log10). Mitochondrial percentage filters were set between 6%-15% . The exact values for each of the 27 10X Genomics datasets are provided in the methods. Each dataset was further individually screened for empty droplets and doublets with Emptydrops and scDblFinder, respectively, and we retained only cells with Emptydrops FDR>0.01 and a doublet score smaller than 0.1. Furthermore, we performed sample-level demultiplexing using deMULTIplex2, whereby multiplets and empty cells were also discarded.<br><br>--<br><br>For the Parse Bio single-cell datasets, standard quality control , preprocessing and doublet removal was done using the manufacturer's software.<br><br>--<br><br>ATAC-seq: We discarded low-quality datasets (NRF<0.65 or PBC1<0.7 or PBC2<1 or FRiP<0.025) and removed peaks overlapping blacklisted regions from ENCODE (http://mitra.stanford.edu/kundaje/akundaje/release/blacklists/hg38-human/hg38.blacklist.bed.gz).
Replication	Stem cell differentiation experiments used for genomics sample collection were repeated 2-7 times on the same human pluripotent stem cell line H7. In our data analysis, we examined the similarity / differences between replicates by low-dimensional projection and used batch as a covariate for appropriate analysis (batch correction and data integration via scVI for scRNA-seq; batch as covariate to the DESeq2 model for ATAC-seq and replicate group for finding markers via the DElegate software's "deseq" method). In addition, we performed a separate batch of differentiation experiments in a separate cell line H9, and similarity was confirmed between cell lines via single-cell label transfer and marker analysis of cognate differentiation stages. This analysis is shown on Supplementary Fig. 8. Replicates of each group can be seen in Supplementary Figs. 2d and 6f (each 10x Genomics run is from a different replicate), Supplementary Fig. 8b (the heatmap shows all replicates based on Parse split-pool scRNA-seq separately), and Fig. 8c (data points from the same day correspond to different replicates).
Randomization	No randomization was performed in this study. Experimental groups were defined by cell line and developmental stage.
Blinding	No blinding was deemed necessary for this study, as all samples were processed equivalently irrespective of sample identity in the computational analyses.

Reporting for specific materials, systems and methods

We require information from authors about some types of materials, experimental systems and methods used in many studies. Here, indicate whether each material, system or method listed is relevant to your study. If you are not sure if a list item applies to your research, read the appropriate section before selecting a response.

Materials & experimental systems

n/a	Involved in the study
☐	☒ Antibodies
☐	☒ Eukaryotic cell lines
☒	☐ Palaeontology and archaeology
☐	☒ Animals and other organisms
☒	☐ Clinical data
☒	☐ Dual use research of concern
☒	☐ Plants

Methods

n/a	Involved in the study
☒	☐ ChIP-seq
☐	☒ Flow cytometry
☒	☐ MRI-based neuroimaging

Antibodies

Antibodies used

Primary antibodies: SOX10 (D5V9L) (Cell Signalling, 89356S,1:500); HOXC9 (Abcam, Ab50839,1:50); MYCN (Santa Cruz, Sc-53993, 1:100); PHOX2B (Santa Cruz, SC-376997, 1:100); MASH1 (ASCL1) (Abcam, Ab211327, 1:100);
Ki-67(Abcam, Ab238020, 1:100); PERIPHERIN (Sigma-Aldrich, AB1530, 1:400). Cleaved Caspase 3 (Asp175) (Cell Signalling, 9661S, 1:400), Phospho-Histone H2A.X (Ser139)(Cell Signalling, 9718S, 1:400).

Secondary antibodies: Goat anti-Mouse Affinipure IgG+IgM (H+L) AlexaFluor 647 (Stratech (Jackson ImmunoResearch) 115-605-044-JIR, Polyclonal 1:500); Donkey anti-Rabbit IgG (H+L) Alexa Fluor 488 (Invitrogen, A-21206, 1:1000).

Validation

SOX10 antibody was validated by the manufacturer for immunofluorescence analysis using SK-MEL-5 cells (positive control) and HeLa cells (negative control), the antibody was validated for flow cytometry using H7 hPSC (Negative control) and hPSC-differentiated Neural crest cells following Frith et al 2018 protocol; HOXC9 antibody was validated by the manufacturer for immunofluorescence analysis using HeLa cells (positive control), for flow cytometry was validated using H7 hPSC (Negative control) and hPSC-differentiated posterior Neuro mesodermal progenitors cells following Frith et al 2018 protocol; MYCN was validated by staining human neuroblastoma cell lines by Izumi et al; PHOX2B was recommended for immunofluorescence analysis by manufacturer, validated with SK-N-SH and IMR-32 neuroblastoma cell lines; MASH1 (ASCL1)(ab211327) antibody was validated by the manufacturer for immunofluorescence analysis on mouse small cell lung cancer as a positive control; MASH1 (ASCL1) (SC-374104) was recommended for immunofluorescence analysis by manufacturer, validated with 293T and SHP-77 cells overexpressing human ASCL1 (MASH1), also used to stain small cell lung cancer in Pongor et al; Ki-67 was validated by manufacturer staining MCF7 (human breast adenocarcinoma cell line) cells as a positive control; PERIPHERIN was validated by the manufacturer staining cultures of rat sensory neurons as positive control; the cleaved caspase-3 antibody was validated by the manufacturer in the context of flow cytometry using Jurkat cells treated with etoposide (compared to untreated cells and cells treated with a nonspecific negative control antibody); the phospho-Histone H2A.X (Ser139) antibody was validated by the manufacturer by immunofluorescence using HeLa cells treated with UV light (vs untreated control).

references:
Izumi H, Kaneko Y. Trim32 facilitates degradation of MYCN on spindle poles and induces asymmetric cell division in human neuroblastoma cells. Cancer Res. 2014;74(19):5620-5630. doi:10.1158/0008-5472.CAN-14-0169

Pongor LS, Schultz CW, Rinaldi L, et al. Extrachromosomal DNA Amplification Contributes to Small Cell Lung Cancer Heterogeneity and Is Associated with Worse Outcomes. Cancer Discov. 2023;13(4):928-949. doi:10.1158/2159-8290.CD-22-0796

Eukaryotic cell lines

Policy information about cell lines and Sex and Gender in Research

Cell line source(s)

We employed the published H7 human embryonic stem cell cell line and its derivatives, as well as the H9 cell line and derivatives. Further information can be found in the Human Pluripotent Stem Cell Registry: https://hpscreg.eu/cell-line/WAe007-A and https://hpscreg.eu/cell-line/WAe009-A

Authentication

hESCs were verified by regularly screening for the expression of typical pluripotency markers such as NANOG, OCT4, and SSEA3/4 by immunofluoreuscence, flow cytometry and qPCR.

Mycoplasma contamination

Cells were regularly monitored for presence of mycoplasma contamination using MYCOSTRIP detection kit from InvivoGen; catalogue number rep-mysnc

Commonly misidentified lines
(See ICLAC register)

None.

Animals and other research organisms

Policy information about studies involving animals; ARRIVE guidelines recommended for reporting animal research, and Sex and Gender in Research

Laboratory animals	Zebrafish (Danio rerio), mutant strain mitfab692/b692; ednrbab140/b140; age for breeding: 6 to 18months, age for experiments: 2-5 days post fertilization.<br><br>NSG mice were obtained from Charles River and enrolled into trial at 6-8 weeks of age. Mice were maintained on a regular diet in a pathogen-free facility on a 12h light/dark cycle with unlimited access to food and water.<br><br>Housing Conditions:<br><br>Humidity: 45 - 65%<br>Temperature: 20 - 24 degrees (Celsius)<br>Lighting: 12h on / 12h off<br>Ventilation: approx. 15 air changes per hour
Wild animals	No wild animals were used in this study.
Reporting on sex	Sex of zebrafish larvae cannot be determined at this early developmental stage and has thus not been considered.<br><br>All mice used in this study were female.
Field-collected samples	This study did not involve samples collected from the field.
Ethics oversight	No ethical approval was needed for larval zebrafish xenograft experiments at the developmental stages used.<br><br>All mouse experiments were approved by The Institute of Cancer Research Animal Welfare and Ethical Review Body and performed in accordance with the UK Home Office Animals (Scientific Procedures) Act 1986, the UK National Cancer Research Institute guidelines for the welfare of animals in cancer research and the ARRIVE (animal research: reporting in vivo experiments) guidelines.

Note that full information on the approval of the study protocol must also be provided in the manuscript.

Plants

Seed stocks	*Report on the source of all seed stocks or other plant material used. If applicable, state the seed stock centre and catalogue number. If plant specimens were collected from the field, describe the collection location, date and sampling procedures.*
Novel plant genotypes	*Describe the methods by which all novel plant genotypes were produced. This includes those generated by transgenic approaches, gene editing, chemical/radiation-based mutagenesis and hybridization. For transgenic lines, describe the transformation method, the number of independent lines analyzed and the generation upon which experiments were performed. For gene-edited lines, describe the editor used, the endogenous sequence targeted for editing, the targeting guide RNA sequence (if applicable) and how the editor was applied.*
Authentication	*Describe any authentication procedures for each seed stock used or novel genotype generated. Describe any experiments used to assess the effect of a mutation and, where applicable, how potential secondary effects (e.g. second site T-DNA insertions, mosiacism, off-target gene editing) were examined.*

Flow Cytometry

Plots

Confirm that:

☒ The axis labels state the marker and fluorochrome used (e.g. CD4-FITC).

☒ The axis scales are clearly visible. Include numbers along axes only for bottom left plot of group (a 'group' is an analysis of identical markers).

☒ All plots are contour plots with outliers or pseudocolor plots.

☒ A numerical value for number of cells or percentage (with statistics) is provided.

Methodology

Sample preparation	Cells were detached and resuspended as single cells using StemPro Accutase Cell Dissociation Reagent (Cat# A1110501, Thermo Fisher Scientific). Standard cell media was used to neutralise accutase. Cells were counted using an haemocytometer. Media containing cells was then centrifuged at 200g for 4mins to pellet cells. Next, 10 million cells/ml were resuspended in 4% PFA at room temperature for 10 minutes. Then cells were washed once with PBS (without Ca2+, Mg2+) and pelleted at 200g. Permeabilisation buffer (0.5% Triton X-100 in PBS with 10% FCS and 0.1%BSA) was added to each sample, followed by incubation at room temperature for 10 minutes. Samples were then washed once with staining buffer (PBS with 10% FCS and 0.1% BSA) and pelleted at 200g. Then samples were resuspended in staining buffer containing pre-

diluted primary antibodies. The samples were left at 4°C on an orbital shaker overnight. Then, the primary antibodies were removed, and samples were washed two times with staining buffer. After washings, staining buffer with pre-diluted secondary antibody was added to the samples and incubated at 4°C for 2 hours. Finally, samples were washed once with staining buffer, resuspended in staining buffer and analysed in the flow cytometer

Instrument	FACSJazz (BD) and CytoFLEX (Beckman Coulter) flow cytometers
Software	BD FACS Software 1.2.0.142 and CytExpert. Data analysis was carried out using FlowJo version 10.8.1
Cell population abundance	n/a
Gating strategy	The initial cell population was identified on a scatter plot of Forward Scatter (FSC) versus Side scatter (SSC) from cells running through the flow cytometer, a gate was drawn around the compact population. Doublets were discriminated using a scatter plot of trigger Pulse width versus SSC. To set the baseline, same cells were stained with secondary antibody only and analysed.

☒ Tick this box to confirm that a figure exemplifying the gating strategy is provided in the Supplementary Information.
